# Subtractive-Dither-Assisted Background Calibration for Linearity Enhancement in Pipelined ADCs for IIoT Applications

**DOI:** 10.3390/s26051632

**Published:** 2026-03-05

**Authors:** Shang Xu, Shuwen Liang, Jinbin Li, Zhenxi Kang, Daolin Zhang, Guoan Wu, Lamin Zhan

**Affiliations:** School of Integrated Circuits, Huazhong University of Science and Technology, Wuhan 430074, China; shangxu0214@hust.edu.cn (S.X.); liangshuwen@hust.edu.cn (S.L.); lijinbin@hust.edu.cn (J.L.); kangzhenxi@hust.edu.cn (Z.K.); dorian_201@hust.edu.cn (D.Z.); wuga5612@hust.edu.cn (G.W.)

**Keywords:** pipelined analog-to-digital converters, background calibration, nonlinearity, dither, linear-feedback shift register, inter-stage amplifier

## Abstract

This paper presents a subtractive-dither-assisted background calibration technique for a 2 GS/s 12 bit pipelined analog-to-digital converter (ADC). A large 7 bit pseudo-random dither is injected in both the flash and the multiplying digital-to-analog converter (MDAC) to decorrelate the differential nonlinearity (DNL) errors caused by the inherent quantization error nonlinearity, capacitor mismatching, and inter-stage amplifier nonlinearity from the input signal. Designed in a 28 nm CMOS process with a 1 V supply, post-layout simulations demonstrate a 10.2 dB improvement in spurious-free dynamic range (SFDR), from 73.8 dB to 84.4 dB, with dithering enabled under a close-to-Nyquist input frequency of 985 MHz. Although the injected dither cannot be completely removed in the digital domain, the proposed ADC exhibits only a 0.5 dB degradation in signal-to-noise-and-distortion ratio (SNDR) for full-scale input, achieving an SNDR of 62.3 dB and an effective number of bits (ENOB) of 10.1 bits. Dithering also improves static performance, with DNL and INL optimized to +0.54/−0.53 LSBs and +0.85/−0.88 LSBs, respectively. Moreover, the proposed dither-based calibration technique introduces an additional power consumption of less than 2 mW.

## 1. Introduction

Driven by the burgeoning demand for data acquisition in the Industrial Internet of Things (IIoT) era, analog-to-digital converters (ADCs) are becoming pivotal in modern intelligent industrial systems. These systems typically require high sample rates, fine resolution, low nonlinearity, low power consumption, and real-time processing [1,2,3,4]. Pipelined ADCs are among the most suitable candidates to meet these demands [5].

Signal-to-noise-and-distortion ratio (SNDR) and spurious-free dynamic range (SFDR) are key performance parameters for ADCs. A high SNDR means less noise and better signal integrity during conversion, while a high SFDR indicates a system’s ability to resolve weak signals in the presence of noise and distortion. For instance, in automated guided vehicles (AGVs), the frequency-modulated continuous wave (FMCW) system is typically employed to measure distances accurately. A high SFDR ensures that the relatively weak reflected signals are not overwhelmed by various types of noise present in industrial environments. One critical challenge in realizing high-SFDR and -SNDR pipelined ADCs is effectively suppressing nonlinearity. Such nonlinearity arises from multiple imperfections, including capacitor mismatch in the sub-ADCs and the multiplying digital-to-analog converters (MDACs), offset of comparators, finite linearity and gain of inter-stage amplifiers, and signal-dependent quantization errors, which collectively degrade the dynamic performance of the converter [6,7,8,9]. For instance, in advanced technology nodes like 28 nm CMOS, the limited intrinsic transistor gain (approximately 15) imposes significant challenges on the design of inter-stage amplifiers requiring high gain, bandwidth, and linearity.

Dithering is an effective technique for mitigating ADC nonlinearity. Precise subtraction of the injected dither signal is mandatory to preserve the SNDR performance. Both theoretical analysis and circuit implementations based on dithering techniques have been reported in the relevant literature [10,11,12,13,14,15,16,17,18,19,20,21,22]. In [10], a small dither is injected into sub-ADCs to calibrate amplifier gain errors. Refs. [11,12] utilize dithering to decouple the nonlinearity from the input signal. Levy provided detailed modeling and analysis of the mechanism by which dithering decorrelates quantization nonlinearity from the input signal [13]. In addition to theoretical studies, dither injection circuits have also been implemented and reported in the recent literature. However, they face certain limitations. For instance, ref. [14] injects a bandwidth-limited dither, which inevitably compromises both the bandwidth and the dynamic range of the input signal. Similarly, ref. [15] proposes a method of injecting dither into the sub-ADC, but this approach reduces the available input range for large-signal sampling. The work [16] introduces capacitive dithering solely within the MDAC. However, it fails to effectively dither the injection-stage inter-stage gain error (IGE). To address these issues, ref. [17] proposed a dual-injection scheme in which dither is applied to both the MDAC and the sub-ADC. By exploiting the cancellation of these two dither paths within the first-stage residue transfer function, the consumption of the redundancy correction range is minimized. Despite these advancements, few studies provide comprehensive details regarding the circuit implementation of dithering.

In this paper, a subtractive-dither-assisted calibration technique is proposed and verified through transistor-level simulations of a 12 bit 2 GS/s pipelined ADC. The technique involves injecting dither into the MDAC and using a current-steering DAC to inject large-amplitude dither into the sub-ADC. This scheme improves linearity and compensates for the redundancy-range penalty from capacitive dither injection in the MDAC. Specifically, a 7 bit dither is injected into the MDAC and a 4 bit dither into the sub-ADC. To minimize redundancy calibration range consumption, the four most significant bits (MSBs) of the MDAC dither share the same control source as the sub-ADC dither. Post-layout simulation results show that the proposed calibration effectively improves the SFDR of the pipelined ADC, with only a negligible degradation in SNDR.

The remainder of this paper is organized as follows. Section 2 analyzes the origins of nonlinearity in pipelined ADCs and describes the circuit implementation details of the proposed large-dither injection scheme. Section 4 presents post-layout simulation results and provides a detailed discussion of the observed performance improvements. Finally, Section 5 concludes this paper.

## 2. Pipelined ADCs with Proposed Subtractive-Dither-Assisted Calibration

### 2.1. Architecture of SHA-Less Pipelined ADCs

**Figure 1:**
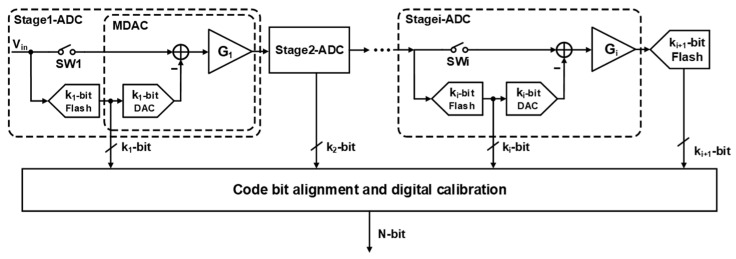
SHA-less pipelined ADC architecture.

The overall architecture of the proposed sample-and-hold amplifier-less (SHA-less) pipelined ADC is depicted in Figure 1. The ADC consists of several cascaded pipelined stages. Each stage integrates a highly linear bootstrapped sampling switch, a *k_i_* bit sub-ADC, and an MDAC. During operation, the sub-ADC directly samples and quantizes the input signal *V_in_* to yield the digital output code *D_i_*. Simultaneously, the MDAC reconstructs the quantized voltage *V_DAC_*, subtracts it from *V_in_*, and amplifies the resulting quantization residue by the residue amplifier (RA) with a gain factor *G_i_*. The amplified residue is then propagated to the subsequent stage for a similar further resolution.

**Figure 2:**
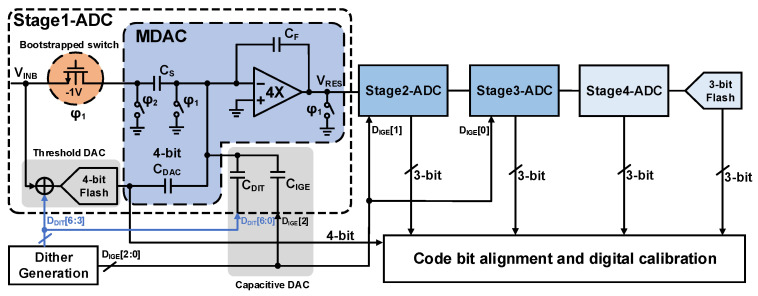
Proposed 7 bit dither injection scheme in 2 GS/s 12 bit pipelined ADC.

In practical implementations, to enable digital error correction through sub-ranging redundancy, the MDAC RA gain *G_i_* is typically scaled down to $2^{k_{i}-1}$ or $2^{k_{i}-2}$. As depicted in Figure 2, a 2 bit redundancy is explicitly allocated to the first stage to absorb comparator offsets. To accommodate the stringent high-speed requirements of the multi-GS/s sampling rate, the sub-ADCs are realized using flash architecture [23].

The operations of digital-to-analog conversion, subtraction, and residue amplification are integrated into a single switched-capacitor block known as the MDAC. As illustrated in Figure 1, the pipelined ADC employs a SHA-less architecture at the front end. Instead of using a dedicated, power-hungry sample-and-hold amplifier (SHA), the input signal is sampled concurrently by both the first-stage MDAC and the parallel flash sub-ADC. This SHA-less configuration significantly alleviates the overall power consumption and eliminates the thermal noise penalty traditionally introduced by a front-end SHA.

### 2.2. A 2 GS/s 12 Bit Pipelined ADC with Dithering

**Figure 3:**
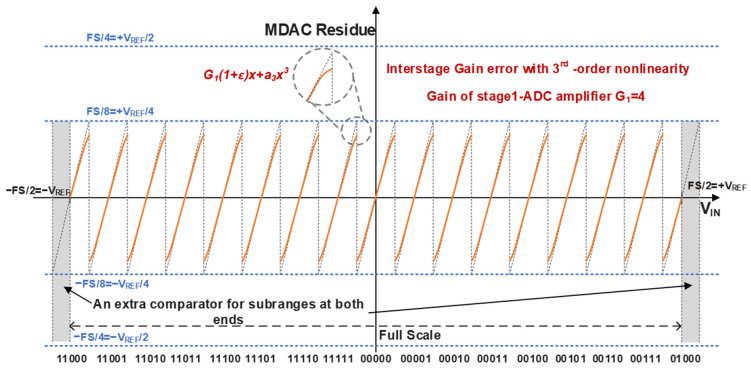
Output residue curve of the first 4 bit sub-ADC.

The pipelined architecture adopted in this work is depicted in Figure 3. The pipeline comprises a 4 bit sub-ADC in the first stage, followed by three cascaded 3 bit sub-ADCs, and ends with a 3 bit flash ADC. Figure 3 also details the circuit implementation of the first stage. The input signal, *V_in_*, is concurrently sampled by both the parallel quantization flash and the MDAC sampling network. The digital codes generated from the coarse quantization then drive the 4 bit DAC capacitor array *C_DAC_*. These codes subtract the corresponding charge from the sampling capacitor *C_S_*. Notably, employing a dedicated *C_DAC_*, rather than reusing *C_S_* for both sampling and DAC operations, effectively suppresses nonlinear charge kickback noise [24].

In the first stage, a large 7 bit dither signal is injected into both the parallel quantizer and the MDAC. This quantizer-side dither linearizes the residual IGE and any inherent nonlinearities in the first-stage residue. Furthermore, the dither introduced into the MDAC propagates down the pipeline, effectively linearizing the DNL errors in the back-end ADC stages. Specifically, to perturb the injection stage, dithering the parallel quantizer is required. To mitigate the performance degradation induced by IGE, the proposed pipeline also incorporates an IGE calibration across the first three pipelined stages.

The residue transfer curve of the first stage is illustrated in Figure 3. By incorporating an additional comparator, a full sub-range is evenly distributed to both ends of the transfer characteristic. The half-sub-range introduced at each end provides sufficient margin to accommodate the proposed 7 bit dither injection without imposing additional constraints on the input signal range. Furthermore, the inter-stage gain of the first-stage amplifier is reduced to 4 rather than the conventional 8 used in standard redundancy calibration. This reduced gain configuration effectively relaxes the output voltage swing constraints. Such a design choice is well suited to the low-voltage and low-power characteristics of the 28 nm CMOS technology, significantly alleviating the design complexity of the high-speed amplifier.

## 3. Analysis of Nonlinearity in Pipelined ADCs

Nonlinearity in pipelined ADCs can be broadly categorized into three types. The first type arises from the inherent quantization error nonlinearity of the ADC, which is fundamentally associated with the discrete nature of the quantization process. The second type is attributed to DNL errors induced by circuit-level nonidealities, including inter-stage amplifier gain nonlinearity, comparator offsets, and capacitor mismatch in the sub-ADCs and MDACs. The third type arises from sampling nonlinearity, mainly due to nonlinear sampling switches, signal-dependent charge injection, and bandwidth limitations in the sampling network. Among these three categories, the first two types of nonlinearity are deterministic and signal-dependent and are therefore amenable to calibration using subtractive-dither-based techniques that effectively decorrelate the nonlinear errors from the input signal. In contrast, sampling nonlinearity is more closely related to device characteristics and circuit design and in most cases cannot be effectively perturbed by dithering. Sampling nonlinearity is closely tied to device characteristics and circuit design methodologies. In most cases, it cannot be effectively randomized by dithering. Therefore, it is typically mitigated through careful front-end circuit design rather than calibration.

### 3.1. Quantization Nonlinearity

**Figure 4:**
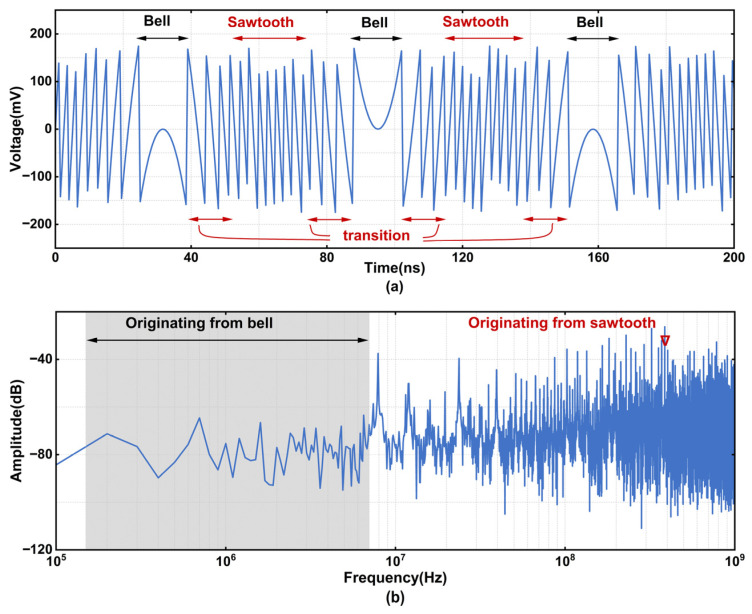
Simulated residue of the first-stage sub-ADC with a 7.89 MHz input signal. (**a**) Time domain wave; (**b**) frequency domain diagram.

Reference [25] provides a detailed analysis of the impact of quantization noise nonlinearity on the output spectrum. Quantization error nonlinearity is an inherent property of ADCs, in that, for a given input signal, the ADC produces a specific digital output code along with a corresponding quantization residue, which can be expressed as

(1) $$V_{in}=D(V_{in})-E_{Q}$$

where *V_in_* denotes the input signal; *D*(*V_in_*) represents the quantized digital output code; and *E_Q_* denotes the quantization error. For a periodic input signal, the corresponding quantization residue also exhibits a periodic pattern, resulting in energy concentration at specific frequencies in the spectrum and degrading SFDR. Figure 4 displays the first-stage residual error waveform and the corresponding FFT at 7.89 MHz. The residual error waveform can be divided into three regions: bell, sawtooth, and transition. The bell region primarily contributes to low-frequency noise, whereas the sawtooth and transition regions contribute to high-frequency noise.

### 3.2. Inter-Stage RA Nonlinearity

**Figure 5:**
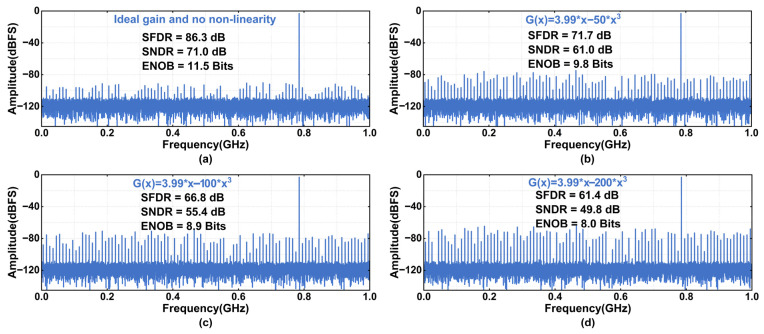
Spectrum of 2 GS/s 12 bit pipelined ADC under different gain nonlinearity conditions. (**a**) Ideal gain with no nonlinearity; (**b**) RA with 0.25% IGE and third-order nonlinear coefficient of 50; (**c**) RA with 0.25% IGE and third-order nonlinear coefficient of 100; (**d**) RA with 0.25% IGE and third-order nonlinear coefficient of 200.

Beyond the inherent quantization noise, the linear IGE and large-signal nonlinearity of the RA are among the primary contributors to the overall nonlinearity in pipelined ADCs [26,27,28,29,30]. Well-designed IGE calibration can achieve an accuracy of 0.25%, indicating that a small residual gain error still exists. In addition, the amplifier also exhibits large-signal nonlinearity errors, as shown in Figure 3. Left unmitigated, these two errors establish a deterministic correlation among the input signal, the output codes, and the quantization residue, which manifests in the frequency domain as energy-concentrated spurious tones. Figure 5 shows the spectral characteristics with residual IGE and different levels of nonlinearity, based on a 2 GS/s, 12 bit pipelined model.

The simulation results in Figure 5 demonstrate a strong correlation between architectural nonidealities and the ADC’s spectral degradation. In the ideal case, the output spectrum exhibits a uniformly distributed noise floor, consistent with power-of-two residue scaling. However, the presence of a residual IGE disrupts this ideal scaling behavior, resulting in the appearance of deterministic spurious tones. As the large-signal nonlinearity of the RA increases, both the number and magnitude of these spurious components grow, indicating a progressive redistribution of signal energy from the fundamental component to higher-order harmonics and intermodulation products. Consequently, the SFDR and SNDR degrade as the nonlinearity increases. This behavior highlights that such deterministic error mechanisms impose a limitation on the dynamic performance of high-resolution pipelined ADCs.

Offset of comparator and capacitor mismatching also serve as significant sources of nonlinearity in pipelined ADCs [31]. These imperfections primarily contribute to DNL errors, which can be categorized as DNL-related nonlinearities.

## 4. Detailed Circuit Implementation of Dithering

**Figure 6:**
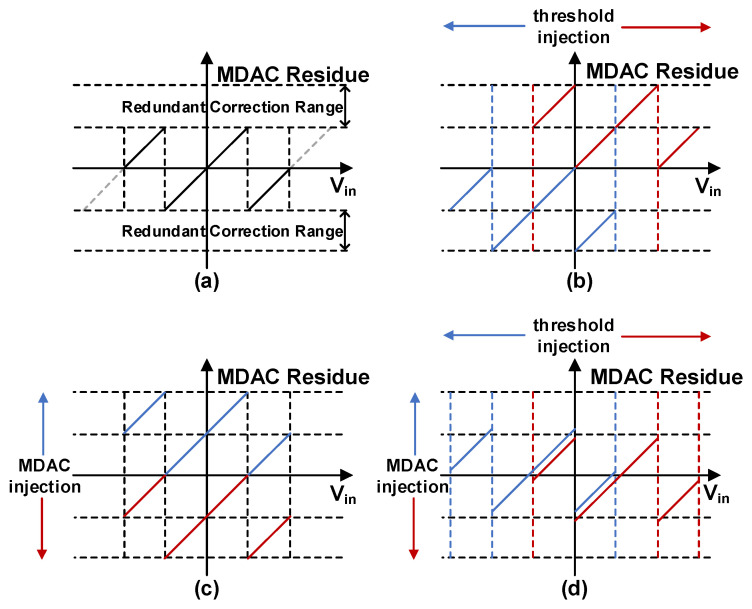
Residue of a simplified 1.5 bit sub-ADC under different dither injection scenarios. (**a**) Without dither injection; (**b**) with only flash dither injection; (**c**) with only MDAC dither injection; (**d**) with simultaneous flash and MDAC dither injection.

This section elaborates on the circuit-level implementation of the proposed dither injection scheme. The 7 bit dither is concurrently injected into both the flash and the MDAC stage. Figure 6 illustrates the first-stage residue transfer curves under various dither injection scenarios. For conceptual clarity, the first stage is simplified to a 1.5 bit sub-stage. Figure 6a depicts the baseline residue characteristic without dither. When dither is injected solely into the flash, as shown in Figure 6b, the residue segments shift along the gain slope, occupying the redundant correction range. In contrast, injecting dither only into the MDAC causes a vertical shift along the y-axis, which similarly consumes the redundancy budget as depicted in Figure 6c. Remarkably, Figure 6d shows that the redundancy occupancy from these two paths can be designed to offset each other through appropriate injection. Theoretically, this dual-path approach consumes no additional redundancy, preserving the full correction range for other circuit nonidealities.

### 4.1. Flash Dither Injection

Compared with dither injection via the MDAC alone, incorporating dither into the flash offers two distinct advantages. On the one hand, as shown in Figure 6, it effectively compensates the redundancy range occupancy induced by the MDAC dither injection, thereby preserving the correction budget. On the other hand, the flash dither enables active perturbation of the MDAC’s inherent nonlinearities within the injection stage itself—a critical capability that MDAC-only injection lacks, as it primarily targets the back-end stages.

**Figure 7:**
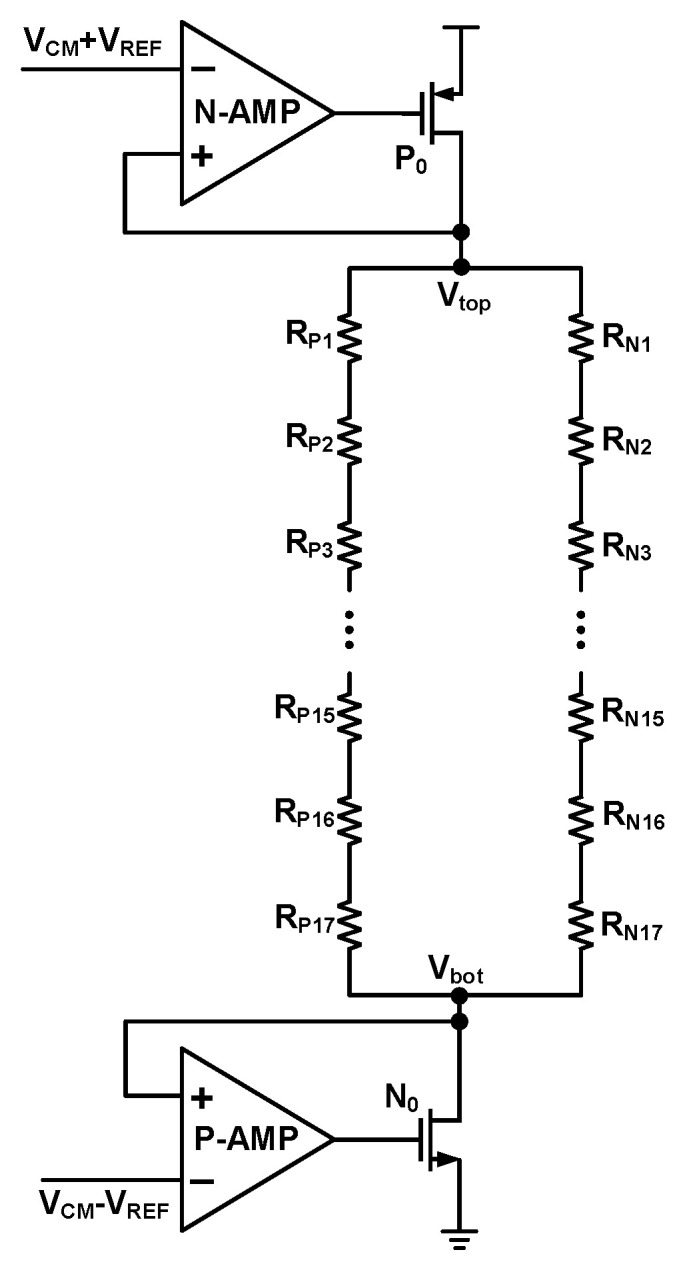
Traditional parallel quantizer reference voltage generation circuit.

Dither injection into the flash is realized by perturbing its reference threshold voltages. Figure 7 illustrates a conventional reference voltage generation circuit for a 4 bit flash. It consists of two operational amplifiers in a unity-gain feedback configuration, which clamp the voltages at the top, *V_top_*, and bottom, *V_bot_*, nodes. A resistive ladder is then connected between these two nodes to derive the required reference threshold voltages.

**Figure 8:**
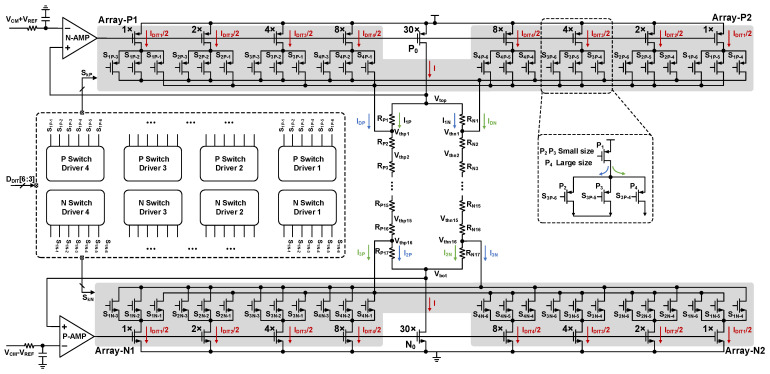
Detailed implementation of the proposed flash dither injection circuit.

Based on the conventional architecture, this work proposes a current-perturbed reference threshold generation scheme, as illustrated in Figure 8.

To effectively utilize the half-sub-ranges at both ends of the residue transfer curve, shown in Figure 3, the flash uses 16 comparators rather than the traditional 15. In this implementation, the current flowing through the main transistor *P*_0_ (sized at 30×) is defined as *I*. The total dither current *I_DIT_*, controlled by a 4 bit digital dither signal, is also designed to equal *I*. This relationship can be expressed as

(2) $$I_{DIT}=I=\sum_{i=1}^{4}I_{DITi}$$

where *I_DITi_* denotes the dither injection current controlled by the *i*-th bit of the digital dither code, and *I_LSB_* represents the least significant current. The value of the resistor ladder in Figure 8 satisfies the following relationship:

(3) $$R_{Pi}=R_{Ni}=R,i\in \{1,2,\ldots ,2^{N}\}$$

When the dither is disabled, the dither injection current is shared equally between *I_DP_* and *I_DN_*, while the main branch current *I* is split evenly between the two resistor columns. In this state, the total threshold reference voltage *V_REF_* satisfies the following:

(4) $$V_{REF}=V_{top}-V_{bot}=\frac{I}{2}•\sum_{i=1}^{17}R_{Pi}+\frac{I}{2}•\sum_{i=2}^{16}R_{Pi}=16I•R$$

Assume that the currents injected into *R_P_*_2_ and *R_N_*_2_ are *I_DP_* and *I_DN_*, respectively, and the currents flowing through *R_P_*_1_, *R_N_*_1_, *R_P_*_17_, and *R_N_*_17_ are denoted by *I*_1*P*_, *I*_1*N*_, *I*_2*P*_, and *I*_2*N*_. Furthermore, let *I*_3*P*_ and *I*_3*N*_ represent the currents flowing from *R_P_*_16_ and *R_N_*_16_ into the dither current sources. The following relationships can be derived for these currents:

(5) $$I=I_{DP}+I_{DN}$$

(6) $$I_{DN}=I_{1P}=I_{2N}=I_{3P}$$

(7) $$I_{DP}=I_{1N}=I_{2P}=I_{3N}$$

The dither current injected into *R_P_*_2_, when expressed in terms of the least significant bit current (*I_LSB_*), can be written as

(8) $$I_{DP}=\sum_{i=1}^{4}PN_{i}•2^{i}I_{LSB}$$

where *PN_i_* denotes the *i*-th bit of the dither digital control code. The voltages *V_P_*_1_ through *V_P_*_16_ and *V_N_*_1_ through *V_N_*_16_ serve as the reference threshold voltages for the differential flash. To implement the half-sub-ranges at both extremities of the residue transfer curve, the dither injection current is not introduced at the *V_top_* and *V_bot_* nodes. Instead, the current is injected through the resistors *R_P_*_2_, *R_N_*_2_, *R_P_*_16_, and *R_N_*_16_, thereby reducing the voltage drop across the resistors at the ends of the ladder. The differential reference threshold voltage can be expressed as

(9) $$V_{thpi}=V_{top}-iI_{DN}•R-(i-1)I_{DP}•R=V_{top}-\frac{i}{16}V_{ref}+I_{DP}•R,i\in \{1,2,\ldots ,2^{N}\}$$

(10) $$V_{thni}=V_{top}-iI_{DP}•R-(i-1)I_{DN}•R=V_{top}-\frac{i}{16}V_{ref}+I_{DN}•R,i\in \{1,2,\ldots ,2^{N}\}$$

where *N* denotes the resolution of the first-stage sub-ADC. When the dither is disabled, *I_DN_* is equal to *I_DP_*, and the two resistor columns equally share both the dither injection current *I_DIT_* and the main branch current *I*. In this balanced state, the differential reference threshold voltage can be expressed as

(11) $$V_{thpi}{|}_{ditoff}=V_{thni}{|}_{ditoff}=V_{top}-(\frac{i}{16}-\frac{1}{32})V_{ref},i\in \{1,2,\ldots ,2^{N}\}$$

The dither-induced threshold voltage shift is obtained by subtracting the reference voltages in the dither-disabled state, as defined in (9) and (10), from the dither-injected values given in (11), as expressed in equation (12). This result is consistent with the threshold voltage variations illustrated in Figure 6.

(12) $$\Delta V_{thpi}=V_{thpi}-V_{thpi}{|}_{ditoff}=I_{DP}•R-\frac{V_{ref}}{32},i\in \{1,2,\ldots ,2^{N}\}$$

(13) $$\Delta V_{thni}=V_{thni}-V_{thni}{|}_{ditoff}=I_{DN}•R-\frac{V_{ref}}{32},i\in \{1,2,\ldots ,2^{N}\}$$

The switching network that controls the dither current is depicted in the dashed box on the right side of Figure 8. In this configuration, transistor *P*1 serves as the dither current sink. Transistors *P*2 and *P*3 are designed with identical dimensions, each being half the size of *P*4. When the current flows in the direction indicated by the blue arrows, the parallel combination of *P*2 and *P*3 is equivalent to *P*4, ensuring consistent current density. Additionally, the dither control arrays, Array-*P*1 and Array-*P*2, utilize a complementary connection to eliminate any inherent circuit asymmetries.

**Figure 9:**
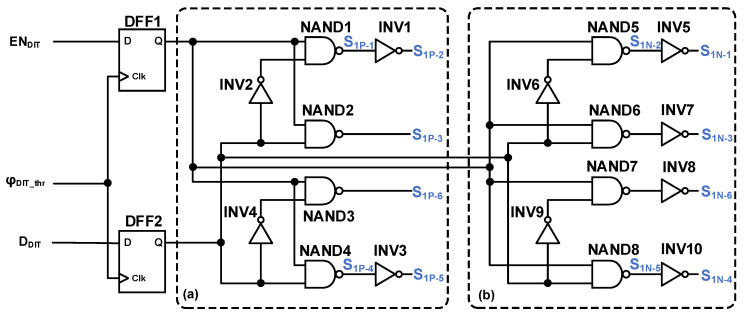
The schematic diagram of dither current switch drivers for (**a**) PMOS switches; (**b**) NMOS switches.

The detailed schematic of the dither current switch driver is illustrated in Figure 9. The core design principle is that, when dither is enabled, the control logic of the two small transistors is identical, which is complementary to that of the large transistor. Conversely, when dither is disabled, only one small transistor is turned on. This configuration ensures that the circuit maintains perfect symmetry regardless of the dither state.

### 4.2. MDAC Dither Injection

**Figure 10:**
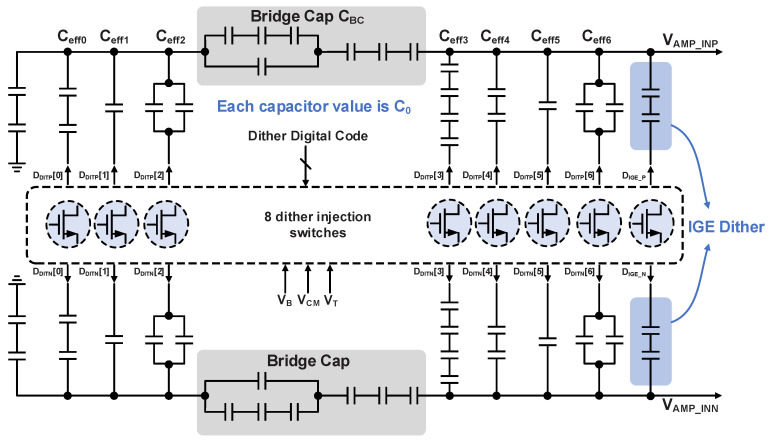
The schematic diagram of MDAC dither injection.

The overall architecture of the MDAC dither injection, as illustrated in Figure 10, comprises a dither injection capacitive DAC array and a driver controller. The dither injection capacitors are binary-weighted from the least significant bit (LSB) to the MSB. To avoid excessively small LSB capacitances that are practically unrealizable in standard fabrication processes, a bridge capacitor is employed to equivalently attenuate the capacitance of the 4 LSBs. This allows the physical capacitors for these LSBs to remain large enough for practical fabrication while still achieving the desired binary scaling through the attenuation provided by the bridge capacitor.

To analyze the dither injection capacitive DAC array, assume all unit capacitors in Figure 9 have a value of *C*_0_. To ensure that the amplitude of the dither generated by the 3rd bit is exactly twice that of the 2nd bit, the bridge capacitor *C_BC_* must satisfy a specific relationship:

(14) $$\frac{(C_{eff0}+\sum_{i=0}^{2}C_{effi})•C_{BC}}{C_{eff0}+\sum_{i=0}^{2}C_{effi}+C_{BC}}=C_{eff3}$$

where *C_effi_* denotes the effective MDAC injection capacitance controlled by the *i*-th dither bit. Solving the above relationship yields

(15) $$C_{BC}=\frac{4}{15}C_{0}$$

**Figure 11:**
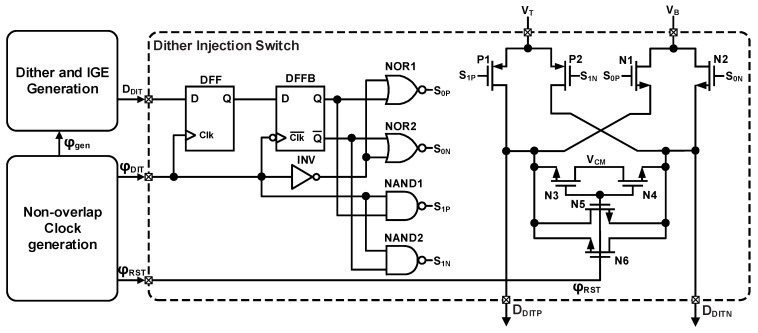
The schematic of switch drivers for MDAC dither injection.

The dither injection switch driver circuit is shown in Figure 11. Its operation relies on a non-overlapping clock generator to produce two phase clocks, φ_DIT_ and φ_RST_. Specifically, φ_RST_ is used to reset the switches to the common-mode voltage, while φ_DIT_ is used to inject the corresponding dither. These two clock phases operate cooperatively to facilitate the complete dither injection process.

### 4.3. Parallel Pseudo-Random Number Generation

In this study, a 23 bit linear-feedback shift register (LFSR) is employed to generate the pseudo-random digital codes required for the dither. The primitive polynomial used to generate these pseudo-random codes is given by

(16) $$P(x)=x^{23}+x^{18}+1$$

The generated bit stream is a maximum-length sequence (MS) with a period of 2^23^ − 1. While this conventional method is well suited for generating single-bit serial pseudo-random sequences, the proposed architecture requires parallel multi-bit random digital codes. If parallel outputs are tapped directly from this single sequence, significant cross-correlation will exist among the output bits, making it difficult to achieve the aforementioned calibration functionality.

**Figure 12:**
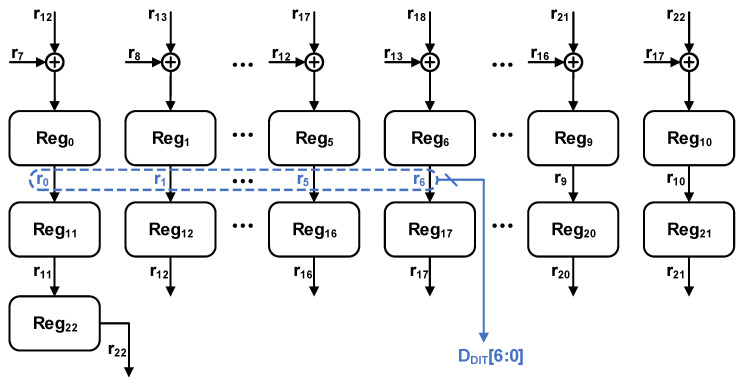
Architecture of parallel 23 bit pseudo-random number generation based on LFSR.

To overcome the aforementioned limitations and achieve superior randomness properties in the parallel digital codes, we adopt the parallel LFSR architecture based on state-space transformation [32,33,34]. The specific generation framework of this parallel LFSR is illustrated in Figure 12, where r_0_ to r_6_ are used for a 7 bit dither in this study.

### 4.4. System Discussion

As indicated by the control logic in Figure 8 and Figure 10, the 4 MSBs of the 7 bit dither signal concurrently control the dither injection in the flash ADC. This shared-control configuration enables the injected flash dither to cancel out the MDAC dither, thereby minimizing the consumption of the available redundancy calibration range. Table 1 summarizes the relationship between the total injection amplitude and the control codes. At full scale, the dither amplitude occupies 127/128 LSBs of the first-stage sub-ADC, almost covering the half-sub-ranges at both ends of the residue curve. The remaining unoccupied 1/128 LSBs corresponds to the two grounded capacitors depicted on the left of Figure 10.

**Figure 13:**
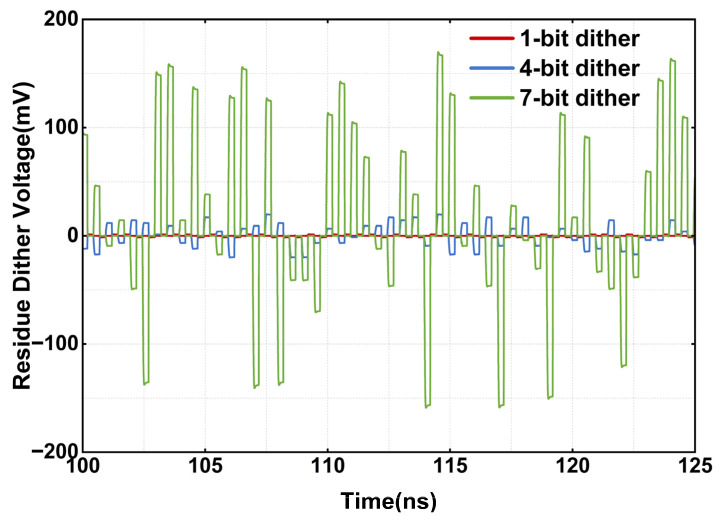
Transient simulation waveforms of first-stage residue at different injection levels.

In the SHA-less architecture, any mismatch between the flash and MDAC sampling paths inevitably consumes additional redundancy correction range. Typical sources of such a mismatch include sampling timing skew and DAC current mirror mismatch. The simulated post-layout residue dither amplitudes corresponding to control codes 0x01, 0x0F, and 0x7F are shown in Figure 13, verifying that the residue outputs are within ±175 mV.

## 5. Post-Layout Simulation Results and Discussion

**Figure 14:**
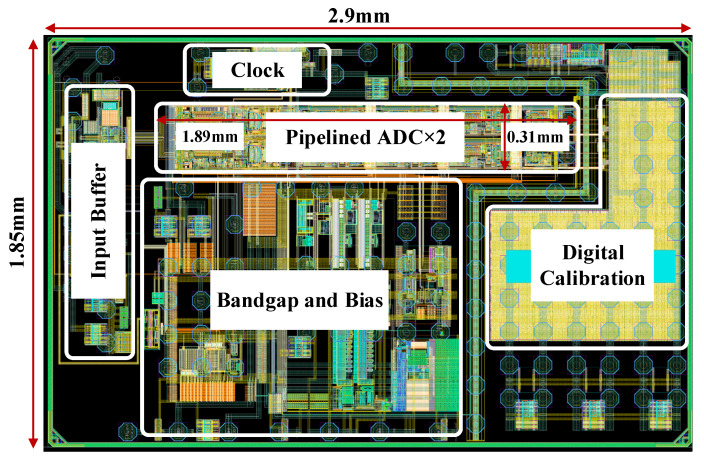
Layout of the pipelined ADC with proposed subtractive-dither-assisted calibration.

The proposed subtractive dither calibration technique was verified through post-layout simulations on a 2 GS/s, 12 bit pipelined ADC implemented in 28 nm CMOS technology. The overall layout of the chip is shown in Figure 14, with dimensions of 2.9 mm × 1.85 mm. The active area of a single-channel pipelined ADC is approximately 1.89 mm × 0.15 mm, comprising the input buffer, biasing circuits, high-speed clock generation, the dual-channel pipelined ADC core, and the digital calibration module. This section primarily presents a comparative analysis of the ADC’s performance with and without calibration. Dynamic performance was evaluated via coherent sampling and a 16,384-point FFT with a rectangular window, where the 0 dBFS full-scale input corresponds to a 1.4 Vpp differential input voltage.

**Figure 15:**
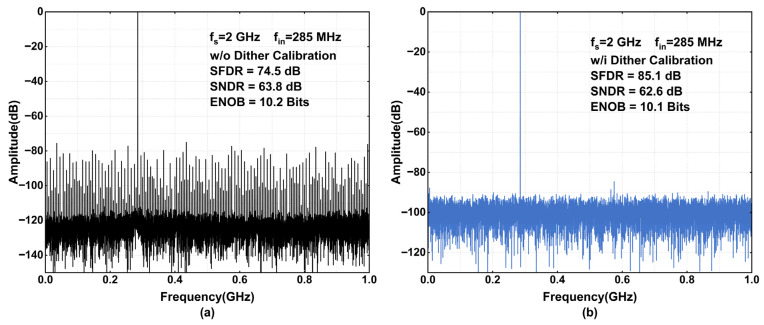
Simulated output spectrum with a 285 MHz sine-wave input. (**a**) Without calibration; (**b**) with proposed subtractive dither calibration.

**Figure 16:**
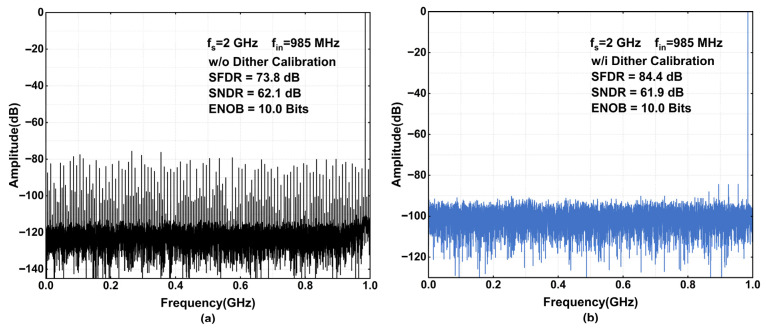
Simulated output spectrum with a 985 MHz sine-wave input. (**a**) Without calibration; (**b**) with proposed subtractive dither calibration.

Figure 15 compares SFDR and SNDR performance with and without calibration at an input frequency of 285 MHz. When calibration is enabled, the SFDR improves significantly from 74.5 dB to 85.1 dB. As observed in Figure 15a, the concentrated spurs and harmonic tones are effectively randomized and spread into the noise floor. It is worth noting that the SNDR suffers a marginal degradation of 0.2 dB. This slight drop is attributed to the residual dither noise, which results from the imperfect subtraction of the analog-injected dither in the digital domain. Figure 16 compares the SFDR and SNDR at an input frequency of 985 MHz, which approaches the Nyquist frequency. With calibration enabled, the SFDR improves from 73.8 dB to 84.4 dB, while the SNDR shows a negligible degradation of only 0.2 dB. This performance corresponds to an ENOB of 10 bits. Collectively, Figure 15 and Figure 16 demonstrate that the proposed dither calibration maintains robust effectiveness across the entire first Nyquist zone.

**Figure 17:**
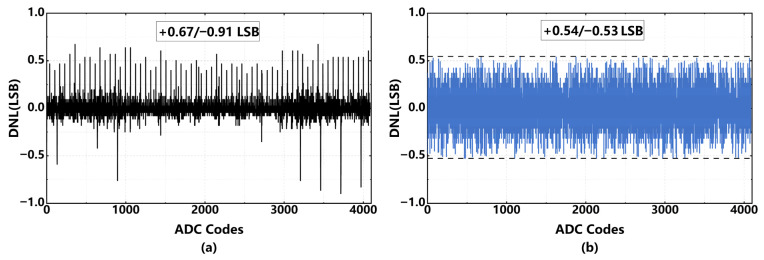
(**a**) Simulated DNL without calibration; (**b**) simulated DNL with proposed 7 bit dither.

**Figure 18:**
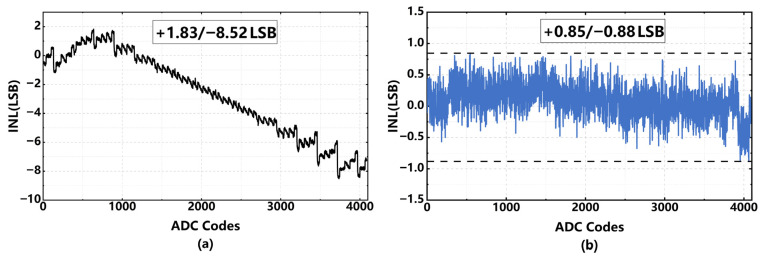
Simulated INL. (**a**) Without calibration; (**b**) with proposed 7 bit dither.

Figure 17 and Figure 18 illustrate the simulated DNL and INL comparisons with and without 7 bit dither. In the uncalibrated state, the ADC exhibits a DNL of +0.67/−0.91 LSBs, with significant errors observed at the sub-range boundaries. Using the proposed 7 bit dither-assisted calibration, the DNL improves to ±0.54 LSBs. Additionally, as depicted in Figure 18a, the uncalibrated INL profile displays a distinct bow shape attributed to inherent nonlinearity. Furthermore, the inter-stage gain error induces pronounced sawtooth-like artifacts towards the upper end of the curve. Upon calibration, the INL is successfully reduced to +0.85/−0.88 LSBs, highlighting the effectiveness of the calibration approach.

**Figure 19:**
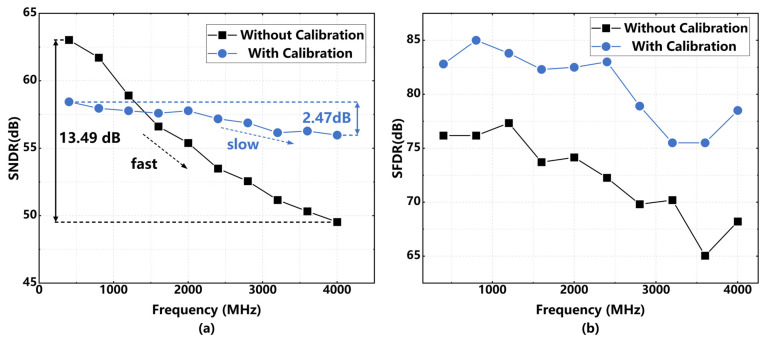
(**a**) SNDR comparison with and without calibration for variable input frequency; (**b**) SFDR comparison with and without calibration for variable input frequency.

Building on this, Figure 19 illustrates the simulated dynamic performance of the ADC as a function of input frequency, with and without calibration. As shown in Figure 19a, the uncalibrated SNDR degrades by 13.49 dB as the input frequency increases to 4 GHz, indicating a strong frequency-dependent performance roll-off. In contrast, the calibrated SNDR demonstrates remarkable stability across the entire Nyquist zone and beyond, with a fluctuation of only 2.47 dB. This flat response verifies that the proposed calibration effectively compensates for bandwidth-limiting errors. Furthermore, Figure 19b shows the SFDR performance, where the calibrated output maintains consistently higher linearity than the uncalibrated case, achieving an improvement of over 10 dB at high input frequencies.

**Figure 20:**
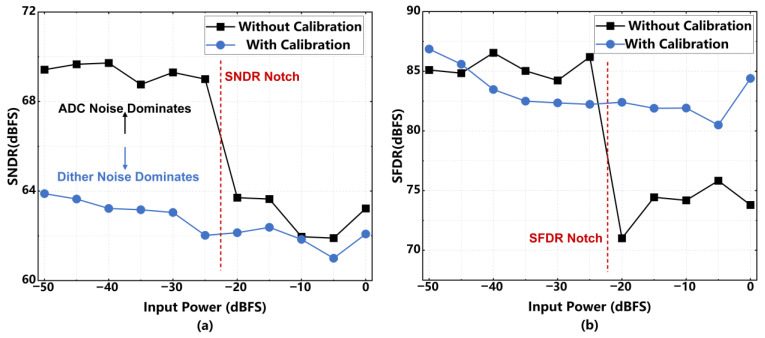
(**a**) SNDR comparison with and without calibration for variable input power; (**b**) SFDR comparison with and without calibration for variable input power.

Figure 20 presents the simulated dynamic performance as a function of input power amplitude. As observed in Figure 20a, the uncalibrated SNDR exhibits a distinctive notch at an input power of approximately −23 dBFS, indicating converter-dominated noise. With the proposed calibration, a highly stable and monotonic response across the full dynamic range is achieved. After calibration, the SNDR improvement is limited by reduced input power, primarily because residual dither noise dominates. Figure 20b demonstrates the substantial benefit in linearity. While the uncalibrated SFDR exhibits a sharp drop at the notch, the calibrated output maintains a robust SFDR, typically greater than 80 dBFS, confirming the calibration’s effectiveness in suppressing harmonic distortion across a wide input power range.

**Figure 21:**
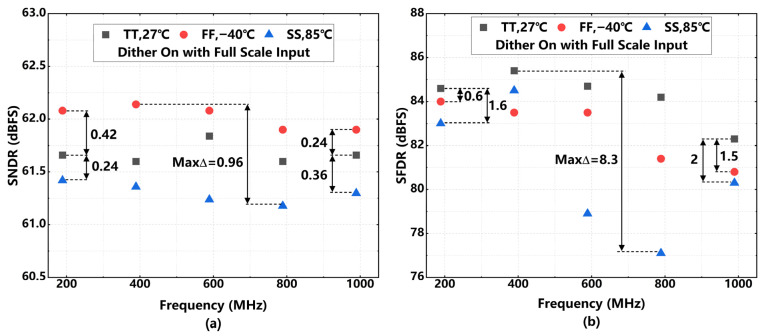
Simulated dynamic performance with proposed 7 bit dither and full-scale input. (**a**) Simulation results of SNDR with PVT variation; (**b**) simulation results of SFDR with PVT variation.

The reliability of the ADC is further verified through process, voltage, and temperature (PVT) corner simulations, as summarized in Figure 21. The design maintains consistent dynamic performance across extreme process corners (FF, SS) and temperature variations (−40 °C to 85 °C). The simulation results reveal that the calibrated SNDR fluctuates by less than 1 dB (Max Δ ≈ 0.96 dB) across the swept frequency range. While the SFDR exhibits slightly higher sensitivity to PVT variations, the calibration scheme successfully preserves the overall linearity, ensuring robust operation in practical implementations.

Table 2 compares the proposed work against other high-speed ADCs. The excellent linearity metrics highlight the effectiveness of the calibration strategy. With a DNL/INL within 0.88 LSBs and an SFDR of 84.4 dB, this work outperforms most comparable designs in spectral purity. While [8] achieves a higher SNDR, it operates at a lower sampling rate of 1 GS/s and uses a 14 bit design resolution. It is noted that the power consumption in this work is higher than in [8] and [26]. This is a trade-off to suppress the noise of sub-ADCs for a higher SFDR and ensure the robustness of PVT corners. Among the 2 GS/s designs, this work maintains high SFDR and ENOB, validating the robustness of the dither-based calibration in suppressing nonlinearities in high-speed pipelined ADCs.

## 6. Conclusions

This paper presents a subtractive-dither-assisted calibration technique to enhance the linearity of pipelined ADCs. By systematically modeling nonlinear sources, the calibration’s operating principle is theoretically analyzed. Post-layout simulation results in a 28 nm CMOS process show that the proposed technique improves the SFDR by more than 10 dB for both low-frequency and near-Nyquist inputs. SNDR degradation is well controlled at less than 0.5 dB with full-scale input. The technique also yields significant improvements in static performance, achieving a DNL and INL of +0.54/−0.53 LSBs and +0.85/−0.88 LSBs, respectively. Furthermore, PVT simulations confirm the robustness and frequency stability, with only 2 mW additional power consumption. This subtractive dither technique is highly effective for high-linearity IIoT applications, such as industrial radar sensing.

## Figures and Tables

**Table 1:** Truth table of dither injection amplitude control.

*D_DIT_* [6:0](Hex)	Active Bits	MDAC Dither Bits	Flash Dither Bits	Dither Range
0x00	0 bit	0 bit	0 bit	0
0x01	1 bit	1 bit	0 bit	±1/256 LSBs *
0x03	2 bit	2 bit	0 bit	±3/256 LSBs
0x07	3 bit	3 bit	0 bit	±7/256 LSBs
0x0F	4 bit	4 bit	1 bit	±15/256 LSBs
0x1F	5 bit	5 bit	2 bit	±31/256 LSBs
0x3F	6 bit	6 bit	3 bit	±63/256 LSBs
0x7F	7 bit	7 bit	4 bit	±127/256 LSBs

* Related to the first 4 bit sub-ADC.

**Table 2:** Performance summary and comparison.

Reference	[8] ^‡^	[26] ^‡^	[27] ^‡^	[12] ^‡^	[5] ^‡^	This Work *
Technology	28 nm	28 nm	28 nm	28 nm	180 nm	**28 nm**
Architecture	Pipeline	Pipelined SAR	Pipeline	TI-Pipeline	Pipeline	**Pipeline**
Resolution	14 bit	12 bit	12 bit	14 bit	16 bit	**12 bit**
Sample Rate	1 GS/s	1.5 GS/s	2 GS/s	5 GS/s	150 MS/s	**2 GS/s**
SNDR/dB @Nyq.	68.2	58.5	58.1	63.0	75.5	**62.0**
ENOB/bits @Nyq.	11.0	9.4	9.4	10.2	12.3	**10.1**
SFDR/dB @Nyq.	85.8	74.5	78.6	80	97.2	**84.4**
DNL/LSBs	−0.65/+0.66	+0.92/−0.74	+0.62/−0.50	N/A	+0.39/−0.34	**+0.54/−0.53**
INL/LSBs	−1.71/+1.48	+2.10/−1.69	+1.90/−0.85	N/A	+2.67/−2.54	**+0.85/−0.88**
Power	15.3 mW	21.3 mW	513.0 mW	1.15 W	415.4 mW	**140.1 mW**
FoMs ^#^	173.3 dB	164 dB	148.0 dB	154.4 dB	158.1 dB	**157.5 dB**

* Post-layout simulation results with TT corner and 27 °C. ^‡^ Measured results. ^#^ FoMs = SNDR + 10 × lg(fsnyq/(2 × Power)), fsnyq is the Nyquist sample rate.

## Data Availability

The datasets produced and/or analyzed in the present study are available from the corresponding author upon reasonable request.

## Abbreviations

The following abbreviations are used in this manuscript:

ADC	Analog-to-digital converter
MDAC	Multiplying digital-to-analog converter
DNL	Differential nonlinearity
SNDR	Signal-to-noise-and-distortion ratio
SFDR	Spurious-free dynamic range
ENOB	Effective number of bits
IIoT	Industrial internet of things
AGVs	Automated guided vehicles
FMCW	Frequency-modulated continuous wave
IGE	Inter-stage gain error
MSBs	Most significant bits
SHA-less	Sample-and-hold amplifier-less
RA	Residue amplifier
SHA	Sample-and-hold amplifier
PDF	Probability density function
LSBs	Least significant bits
LFSR	Linear-feedback shift register
MS	Maximum-length sequence
PVT	Process, voltage, and temperature

## References

[1] Zhang J., Sun T., Huang Z., Tao W., Wang N., Tian L., Zhu Y., Wang H. (2025). The Design of a Low-Power Pipelined ADC for IoT Applications. Sensors.

[2] Ali A.M.A., Morgan A., Dillon C., Patterson G., Puckett S., Bhoraskar P., Dinc H., Hensley M., Stop R., Bardsley S. (2010). A 16-Bit 250-MS/s IF Sampling Pipelined ADC with Background Calibration. IEEE J. Solid-State Circuits.

[3] Zhang Z., Hu Y., Lang L., Dong Y. (2024). A 16 Bit 125 MS/s Pipelined Analog-to-Digital Converter with a Digital Foreground Calibration Based on Capacitor Reuse. Electronics.

[4] Ali A.M.A., Dillon C., Sneed R., Morgan A.S., Bardsley S., Kornblum J., Wu L. (2006). A 14-Bit 125 MS/s IF/RF Sampling Pipelined ADC with 100 dB SFDR and 50 Fs Jitter. IEEE J. Solid-State Circuits.

[5] Zhu C., Liang R., Lin J., Wang Z., Li L. (2019). Analysis and Design of a Large Dither Injection Circuit for Improving Linearity in Pipelined ADCs. IEEE Trans. Very Large Scale Integr. (VLSI) Syst..

[6] Linnhoff S., Buballa F., Reinhold M., Spanl R., Sippel E., Gerfers F. A 12-Bit 6-GS/s Time-Interleaved SAR ADC with On-Chip Mismatch Calibration in 28nm CMOS Technology. Proceedings of the 2025 IEEE Radio Frequency Integrated Circuits Symposium (RFIC).

[7] Cao Y., Zhang M., Zhu Y., Martins R.P., Chan C.-H. (2024). A 12-GS/s 12-b 4× Time-Interleaved ADC Using Input-Independent Timing Skew Calibration with Global Dither Injection and Linearized Input Buffer. IEEE J. Solid-State Circuits.

[8] Cao Y., Shen Y., Liu S., Han H., Liang H., Dang L., Li D., Ding R., Zhu Z. 24.2 A 14b 1GS/s Single-Channel Pipelined ADC with A Parallel-Operation SAR Sub-Quantizer and A Dynamic-Deadzone Ring Amplifier. Proceedings of the 2025 IEEE International Solid-State Circuits Conference (ISSCC).

[9] Wang P., Wu N., Li F., Wang Z. (2025). A 1-GS/s 12-Bit Pipelined-SAR ADC with Dither-Based Background Calibration of Interstage Gain and Comparator Offset in 28-nm CMOS. IEEE Trans. Circuits Syst. I Regul. Pap..

[10] Ali A.M.A., Dinc H., Bhoraskar P., Dillon C., Puckett S., Gray B., Speir C., Lanford J., Brunsilius J., Derounian P.R. (2014). A 14 Bit 1 GS/s RF Sampling Pipelined ADC with Background Calibration. IEEE J. Solid-State Circuits.

[11] Chen L., Cao Y., Ling L., Liu S., Han H. (2025). Metastable-Dither-Based Digital Background Calibration of Interstage Gain Nonlinearity in Pipelined SAR ADC. IEEE Trans. Very Large Scale Integr. (VLSI) Syst..

[12] Ali A.M.A., Dinc H., Bhoraskar P., Puckett S., Morgan A., Zhu N., Yu Q., Dillon C., Gray B., Lanford J. A 14-Bit 2.5GS/s and 5GS/s RF Sampling ADC with Background Calibration and Dither. Proceedings of the 2016 IEEE Symposium on VLSI Circuits (VLSI-Circuits).

[13] Levy B.C. (2013). A Study of Subtractive Digital Dither in Single-Stage and Multi-Stage Quantizers. IEEE Trans. Circuits Syst. I Regul. Pap..

[14] Gonzalez-Diaz V.R., Garcia-Andrade M.A., Flores-Verdad G.E., Maloberti F. (2010). Efficient Dithering in MASH Sigma-Delta Modulators for Fractional Frequency Synthesizers. IEEE Trans. Circuits Syst. I Regul. Pap..

[15] Fetterman H.S., Martin D.G., Rich D.A. (1999). CMOS Pipelined ADC Employing Dither to Improve Linearity. Proceedings of the Proceedings of the IEEE 1999 Custom Integrated Circuits Conference (Cat. No.99CH36327).

[16] Rakuljic N., Galton I. (2013). Suppression of Quantization-Induced Convergence Error in Pipelined ADCs with Harmonic Distortion Correction. IEEE Trans. Circuits Syst. I Regul. Pap..

[17] Devarajan S., Singer L., Kelly D., Pan T., Silva J., Brunsilius J., Rey-Losada D., Murden F., Speir C., Bray J. (2017). A 12-b 10-GS/s Interleaved Pipeline ADC in 28-Nm CMOS Technology. IEEE J. Solid-State Circuits.

[18] Jiang Z., Wu D., Guo X., Jia H., Chen P., Wu F. A 1.5GS/s Single-Channel Pipelined ADC with Large Dither Technique. Proceedings of the 2023 8th International Conference on Integrated Circuits and Microsystems (ICICM).

[19] Gu M., Zhong Y., Jie L., Sun N. 24.1 A 12b 3GS/s Pipelined ADC with Gated-LMS-Based Piecewise-Linear Nonlinearity Calibration. Proceedings of the 2025 IEEE International Solid-State Circuits Conference (ISSCC).

[20] Wang P., Li F., Wang Z. (2025). A Single-Channel 8-Bit 1.6-GS/s Alternate-Comparator SAR ADC with Dither-Based Background Offset Calibration in 28-Nm CMOS. IEEE Trans. Circuits Syst. I Regul. Pap..

[21] Wu F., Guo X., Jia H., Wu X., Li Z., He B., Wu D., Liu X. (2023). A 12-Bit 2 GS/s Single-Channel High Linearity Pipelined ADC in 40 Nm CMOS. Micromachines.

[22] Meng L., Zhao M., Tan Z. SQNR Improvement of Incremental Zoom ADCs with Raised-Order CoI Filter and Dither Injection. Proceedings of the 2025 IEEE International Symposium on Circuits and Systems (ISCAS).

[23] Zhang H., He B., Guo X., Wu D., Liu X. (2023). A 1-GS/s 12-Bit Single-Channel Pipelined ADC in 28-Nm CMOS with Input-Split Fully Differential Ring Amplifier. IEEE Trans. Very Large Scale Integr. (VLSI) Syst..

[24] Devarajan S., Singer L., Kelly D., Decker S., Kamath A., Wilkins P. (2009). A 16-Bit, 125 MS/s, 385 mW, 78.7 dB SNR CMOS Pipeline ADC. IEEE J. Solid-State Circuits.

[25] Pan H., Abidi A.A. (2004). Spectral Spurs Due to Quantization in Nyquist ADCs. IEEE Trans. Circuits Syst. I Regul. Pap..

[26] Shen Y., Liu S., Cao Y., Han H., Liang H., Dong Z., Li D., Ding R., Zhu Z. (2025). A 12-Bit 1.5-GS/s Single-Channel Pipelined SAR ADC with a Pipelined Residue Amplification Stage. IEEE J. Solid-State Circuits.

[27] Ni Y., Liu L., Zhang Y., Zhu T. (2025). A 12-Bit 2-GS/s Pipeline ADC in 28-Nm CMOS with Linear-Error Self-Calibration. IEEE Trans. Very Large Scale Integr. (VLSI) Syst..

[28] Devarajan S., Gutmann R.J., Rose K. (2007). A 12-Bit 65 MS/s Pipeline A/D Converter in 0.18 Μm SiGe BiCMOS. 2007 IEEE Bipolar/BiCMOS Circuits and Technology Meeting.

[29] Wang Q., Peng X., Lu Z., Peng Y., Hu Z., Tang H. Digital Background Calibration Techniques for Interstage Gain Error and Nonlinearity in Pipelined ADCs. Proceedings of the 2024 IEEE International Symposium on Circuits and Systems (ISCAS).

[30] Liu Y., Hao M., Xu H., Gao X., Zheng H. (2025). Ensembling a Learned Volterra Polynomial with a Neural Network for Joint Nonlinear Distortions and Mismatch Errors Calibration of Time-Interleaved Pipelined ADCs. Sensors.

[31] Thai H.-H., Pham C.-K., Le D.-H. (2022). Design of a Low-Power and Low-Area 8-Bit Flash ADC Using a Double-Tail Comparator on 180 Nm CMOS Process. Sensors.

[32] Hu G., Sha J., Wang Z. (2017). High-Speed Parallel LFSR Architectures Based on Improved State-Space Transformations. IEEE Trans. Very Large Scale Integr. (VLSI) Syst..

[33] Katti R.S., Ruan X., Khattri H. (2006). Multiple-Output Low-Power Linear Feedback Shift Register Design. IEEE Trans. Circuits Syst. I: Regul. Pap..

[34] Kuehnel R., Theiler J., Wang Y. (2006). Parallel Random Number Generators for Sequences Uniformly Distributed over Any Range of Integers. IEEE Trans. Circuits Syst. I Regul. Pap..

